# Myocyte-specific overexpressing HDAC4 promotes myocardial ischemia/reperfusion injury

**DOI:** 10.1186/s10020-018-0037-2

**Published:** 2018-07-17

**Authors:** Ling Zhang, Hao Wang, Yu Zhao, Jianguo Wang, Patrycja M. Dubielecka, Shougang Zhuang, Gangjian Qin, Y Eugene Chin, Race L. Kao, Ting C. Zhao

**Affiliations:** 1 Department of Surgery, Boston University Medical School, Roger Williams Medical Center, 50 Maude Street, Providence, RI 02908 USA; 2 Department of Emergency Medicine, Department of Medicine, Rhode Island Hospital, Brown University, Providence, RI USA; 3 Feinberg Cardiovascular Research Institute, Northwestern University Feinberg School of Medicine, Chicago, USA; 4 Key Laboratory of Stem Cell Biology, Institutes of Health Sciences, Shanghai Institutes for Biological Sciences, Chinese Academy of Sciences, Shanghai, China; 5 Department of Surgery, East Tennessee State University, Johnson City, TN USA

**Keywords:** Histone deacetylase4 (HDAC4), Ischemia/reperfusion, Myocardial function

## Abstract

**Background:**

Histone deacetylases (HDACs) play a critical role in modulating myocardial protection and cardiomyocyte survivals. However, Specific HDAC isoforms in mediating myocardial ischemia/reperfusion injury remain currently unknown.

We used cardiomyocyte-specific overexpression of active HDAC4 to determine the functional role of activated HDAC4 in regulating myocardial ischemia and reperfusion in isovolumetric perfused hearts.

**Methods:**

In this study, we created myocyte-specific active HDAC4 transgenic mice to examine the functional role of active HDAC4 in mediating myocardial I/R injury. Ventricular function was determined in the isovolumetric heart, and infarct size was determined using tetrazolium chloride staining.

**Results:**

Myocyte-specific overexpressing activated HDAC4 in mice promoted myocardial I/R injury, as indicated by the increases in infarct size and reduction of ventricular functional recovery following I/R injury. Notably, active HDAC4 overexpression led to an increase in LC-3 and active caspase 3 and decrease in SOD-1 in myocardium. Delivery of chemical HDAC inhibitor attenuated the detrimental effects of active HDAC4 on I/R injury, revealing the pivotal role of active HDAC4 in response to myocardial I/R injury.

**Conclusions:**

Taken together, these findings are the first to define that activated HDAC4 as a crucial regulator for myocardial ischemia and reperfusion injury.

**Electronic supplementary material:**

The online version of this article (10.1186/s10020-018-0037-2) contains supplementary material, which is available to authorized users.

## Background

Histone deacetylases (HDACs) are a group of enzymes that regulate gene expression by the modulation of their interactions with chromatin through the deacetylation of histones. The acetylation and deacetylation of chromatin histones are considered to be critical in the regulation of transcription in in the biological responses. Acetylation of histone is caused by histone acetyl transferase, which leads to nucleosomal relaxation and altered transcriptional activation. In contrast, histone deacetylase result in deacetylation and transcriptional repression (Turner 2000; McKinsey 2012).

Since the identification of HDAC 1 (Hassig et al. 1998), 18 HDACs have been identified and were classified into three distinct groups (Verdin et al. 2003). Class I HDACs consist of HDACs 1, 2, 3, and 8. Class II HDACs are further divided into the following: IIa (HDACs 4, 5, 7 and 9) and IIb (HDACs 6 and 10). It is notable that both HDAC 4 and HDAC 5 are highly expressed in the myocardium, brain and skeletal muscles, which indicates that both HDACs are important in modulating the biological function of these organs. (Fischle 1999; Grozinger et al. 1999; Wang et al. 1999). Class III HDACs were identified on the basis of sequence similarity with Sir, which includes SIRT1–7 and Sir2.

Recent studies have demonstrated that HDACs play an important role in the development of myocardial hypertrophy and ischemic injury (Antos et al. 2003; Kee et al. 2006; Kong et al. 2006; Haberland et al. 2009; Granger et al. 2008). Inhibition of HDAC with chemical inhibitor trichostatin A attenuated cardiomyocyte hypertrophy. Likewise, pharmacological inhibition of HDACs suppressed myocardial hypertrophy and improved cardiac performance in vivo (Kong et al. 2006; Haberland et al. 2009). Furthermore, HDAC inhibition was found to be closely associated with the attenuation of myocardial ischemia and reperfusion injury in mice (Granger et al. 2008). More importantly, our extensive studies in animal models suggest that pharmacological inhibition of HDAC is considered to be one of the most important signals to reduce myocardial ischemia and reperfusion injury and improve cardiac performance (Zhao et al. 2007; Zhang et al. 2012a; Zhang et al. 2012b; Zhang et al. 2010). Additionally, we also demonstrated that HDAC inhibition or genetic inhibition of specific HDAC4 promoted myocardial repair through stimulating cardiac progenitor cells (Zhang et al. 2012a; Zhang et al. 2012b). Notably, we have found that HDAC inhibition increased the survival of embryonic stem cells through the reduction and degradation of HDAC4 isoform (Chen et al. 2011).

It is generally recognized that class II HDACs are critical to modulate cardiac injury, hypertrophy and development, but HDAC4 demonstrates very little activity. Most of the studies only focused on defining the function of the magnitude of HDAC expression rather than the activation of HDAC4, especially *activated* HDAC4, in modulating myocardial function. It is critical to identify the function of activated HDAC4 in the heart. These evidences indicate that HDAC4 is one of the most important class II HDACs in the heart and muscle and plays a critical role in modulating cardiac development, ischemic injury, and hypertrophy. In the present study, we created cardiac HDAC4 transgenic mice in which HDAC4 was activated to determine how active HDAC4 modulates myocardial injury. This will provide new insight into understanding the functional role of activated HDAC4 in heart disease.

## Materials and methods

### Generation of cardiac specific active HDAC4 mice

Creation of the mice carried out in Boston University transgenic core facility. A cDNA encoding an activated HDAC4 was cloned into an expression vector encoding alpha-myosine heavy chain (the α-MHC promoter, 5.4 kb), a cardiomyocyte-specific promoter at the multiple cloning site. After ligation, the construct was purified and verified by restriction enzyme digestion and sequencing. Transgenic mice were generated by microinjection of the α-MHC-HDAC4 DNA construct into fertilized FVB/n mouse eggs F_1_ eggs. Founder mice and transgenic expression of HDAC4 were identified by analysis of genomic DNA with primer A (5-CCTCGTTCCAGCTGTGGT-3); a sense primer specific to MHC promoter exon 2) and antisense primer B (5-AGCGCCAGGAGCTCCTGCTGC-3); specific to HDAC4 cDNA. The protocol for the animal experiments in this study was approved by IACUC, which is fully in agreement with the guidance for the Care and Use of Laboratory Animals published by the US National Institutes of Health.

### Reagents and antibodies

Trichostatin A, 3-[4,5-dimethylthiazol-2-yl]-2,5- diphenyltetrazolium bromide (MTT) and 4,6-Diamidino-2-phenylindole (DAPI) were obtained from Life Technologies (Grand Island, NY). Primary antibodies including HDAC4 rabbit polyclonal and β-actin antibodies (Cell Signaling ^Tm^ (Beverly, MA), and primary active caspase 3 were purchased from Abcam (Cambridge, MA). SOD-1 and LC3 poly clonal primary antibodies was purchased from Santa Cruz biotechnology (Dallas, Texas). All chemicals for perfused hearts were purchased from Aldrich-Sigma (St. Louis, Missouri).

### Langendorff isolated heart perfusion and functional measurement

The methodologies of Langendorff perfused system, ventricular function detection, and infarct size measurement has been described previously (Zhao et al. 2007). Briefly, adult male mice were anesthetized with a lethal intraperitoneal injection (i.p.) of sodium pentobarbital (120 mg/kg). The hearts were rapidly isolated and kept in ice-cold Krebs-Henseleit buffer. The isolated hearts were then cannulated through the ascending aorta in the isovolumetrically perfused system (Langendorff method) for retrograde perfusion using oxygenated Krebs-Henseleit buffer. They were then cannulated via the ascending aorta for retrograde perfusion by the Langendorff method using Krebs-Henseleit buffer containing 2.5 mmol/L of CaCl_2_2H_2_O. During the course of the retrograde perfusion, Krebs-Henseleit buffer was continuously aerated with 95%O_2_:5%CO_2_ to maintain the value of pH of Krebs-Henseleit buffer at 7.4. The Langendorff system was maintained at 37 °C, and the perfusion pressure was adjusted at a constant pressure of 55 mmHg. A water-filled latex balloon, attached to the tip of polyethylene tubing, was then inflated sufficiently to provide a left ventricular end-diastolic pressure (LVEDP) of about 10 mmHg. Left ventricular function was assessed by inserting a water-filled latex balloon into the left ventricle, which was connected to a pressure transducer and recorded through Power Lab recording system (ADInstruments, Bella Vista, AUSTRALIA). Ventricular functional parameters were measured, which include left ventricular end-diastolic pressure (LVEDP), left ventricular systolic pressure (LVSP), left ventricular developed pressure (LVDP), rate pressure product (RPP), heart rate (HR), and coronary flow (CF).

### Experimental protocol for myocardial I/R injury

Mice about 2 month old were randomized into four experimental groups that underwent the following treatments, as shown in the experimental protocol**.** *Control wild-type* and *α-MHC-HDAC4* mice were subjected to 30 min of stabilization and 30 min of ischemia followed by 30 min of reperfusion. To examine the contribution of HDAC4 to cardioprotection elicited by HDAC inhibition, we treated animals with HDAC inhibitor to determine whether HDAC inhibitor was able to attenuate the detrimental effects of HDAC4 over-expression in the heart. We utilized an established preconditioning protocol. Control wild-type and α-MHC-HDAC4 mice were treated (i.p. injection) with TSA 24 h before ischemia. Animals were divided into two additional groups: *TSA + Wild* type mice (*n* = 5), Wild type mice were injected with TSA (0.1 mg/kg ip); *TSA + α-MHC-HDAC4* mice (n = 5), α-MHC-HDAC4 mice were injected with TSA (0.1 mg/kg ip). Twenty-four hours later, the hearts were subjected to 30 min of ischemia followed by 30 min of reperfusion.

### Measurement of myocardial infarction

Tetrazolium chloride **(**TTC) staining was employed to detect infarct size. After Langendorff perfusion, the hearts were then frozen in the refrigerator for a short period. Then, the frozen hearts were cut from apex to base into 1 mm thick slices. These slices were then placed in 10% TTC for 20 min. The cardiac sections were fixed in paraformaldehyde (4%) for photography. NIH ImageJ software was utilized to measure the area of viable and dead portion of tissues. The infarct size of each heart was determined and shown as the percentage of risk area, defined as the sum of total ventricular area minus cavities (Zhao et al. 2007).

### Echocardiographic measurement of cardiac function

Cardiac functions of wild type and α-MHC-HDAC4 transgenic mice were assessed using echocardiographic measurements. Ventricular parameters include left ventricular internal dimension in end and systole (LVID;d and LVID;s); fractional shortening (FS) and ejection fraction (EF), which were described previously (Zhang et al. 2017).

### Electrophoresis and western blot analysis

Protein extraction and western blot for analysis of protein expression were conducted as described as before (Zhao et al. 2007). In brief, myocardial tissues were isolated and then homogenized in cold lysis buffer containing protease inhibitor cocktails (Calbiochem, Billerica, MA). The protein lysates were subjected to centrifugation at 12,000 g at 4 °C for 15 min. The supernatant of these samples were then collected, and the protein concentration of the samples were determined using a Micro BCA Assay Kit (Thermo Scientific, Rockford, IL). The samples (50 μg/per lane) were loaded and run on SDS polyacrylamide gels at a constant voltage 100 V and transferred to polyvinylidene difluoride membrane at 24 V (PVDF). The PVDF was blocked in non-fat dry milk (5%) at room temperature for 1 h followed by incubation with individual primary antibodies, their respective polyclonal antibodies. Protein signals were then visualized by incubation with anti-rabbit horseradish peroxidase-conjugated secondary antibody and developed with ECL Chemiluminescence detection reagent (Amersham Pharmacia Biotech).

### Immunohistochemistry

The cardiac tissues were fixed with buffered paraffin and then embedded samples were cut into 10 μm-thick sections. Tissue sections were de-paraffinized in xylene and then rehydrated at decreasing concentrations of ethanol, which was described in our previous protocol (Zhang et al. 2012a). Active caspase 3 was used to assess for apoptotic signals in the heart. LC-3 was used to determine the signal of autophagy in myocardium. Positive signals in term of active caspase 3 and LC-3 from cardiac sections were counted from 3 to 5 randomized fields. A detailed methodology of immunostaining for detection of active caspase-3 and LC3 was carried out as described in our previous protocol (Zhang et al. 2012a).

### Measurement of HDAC activity

Measurement of cardiac HDAC activity was carried out by using by using a colorimetric HDAC activity assay kit (BioVision, Mountain View, CA).

### Statistical analysis

All data, including ventricular function, infarct size, protein density, and immunostaining signals are expressed as mean ± SE. Differences among the groups were analyzed by one-way analysis of variance (ANOVA) followed by Bonferroni correction. Student’s unpaired *t* test was conducted to compare the difference between groups. *p* < 0.05 was considered to be a significant difference between groups.

## Results

### Characterization of cardiac-specific HDAC4 mice

**Fig. 1:**
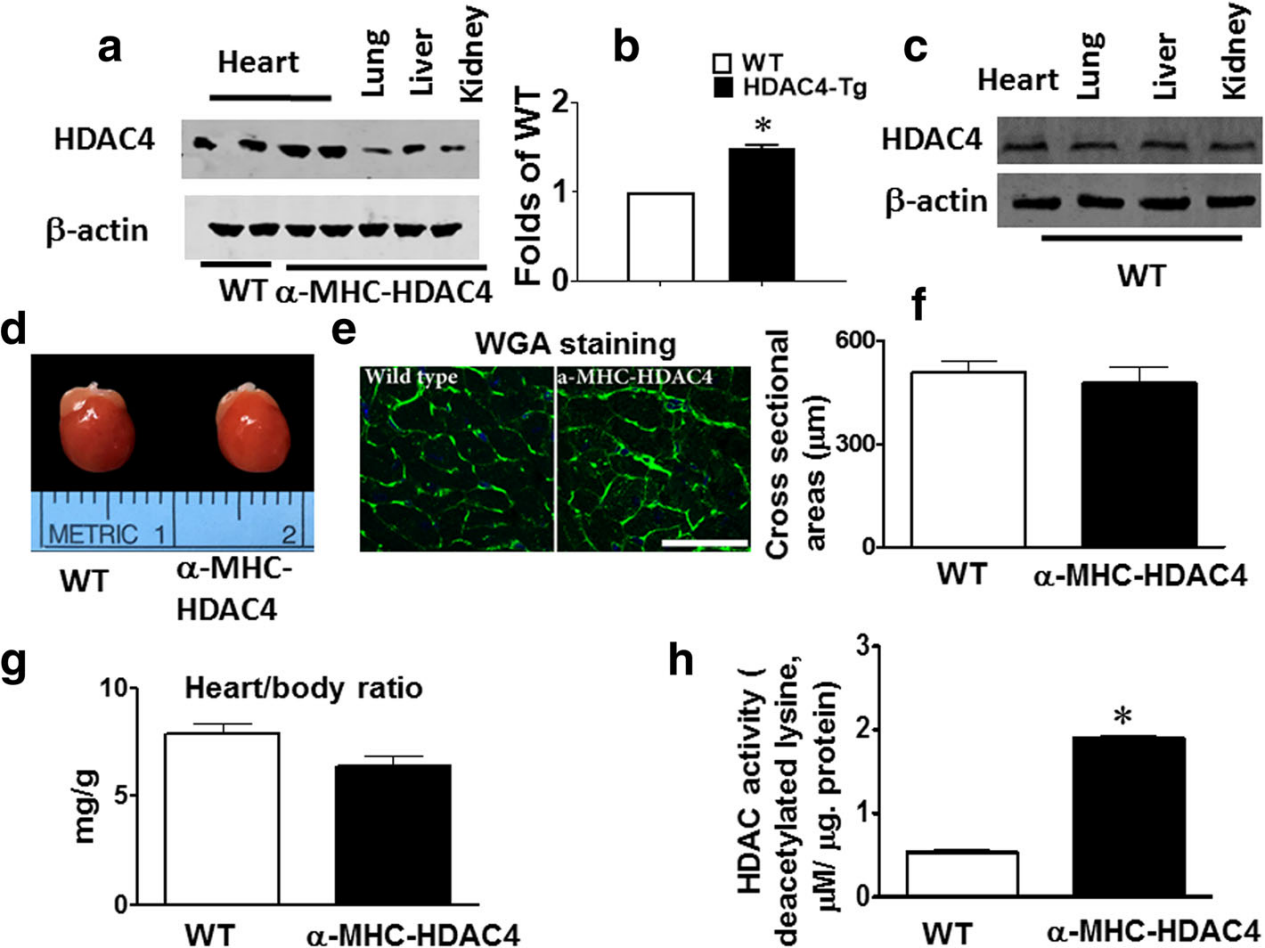
Generation and characterization of cardiac myocyte-specific HDAC4 transgenic mice. **a** HDAC4 over-expression in α-MHC-HDAC4 mice and wild type mice; **b** Densitometric analysis of HDAC4 proteins from the heart; **c** HDAC4 proteins in heart and other organs in wild type mice (scale bar = 50 μm); **d** Isolated hearts from α-MHC-HDAC4 mice and wild type mice; **e** WGA staining of cardiomyocytes of α-MHC-HDAC4 mice and wild type mice; **f** Cardiomyocyte sizes in α-MHC-HDAC4 mice and wild type mice; **g** Heart/body weight ratio from α-MHC-HDAC4 mice and wild type mice; **h** HDAC activity measured from α-MHC-HDAC4 mice and wild type mice; values are present as Mean Values representing mean ± SE (*n* = 3–5/per group)

**Fig. 2:**
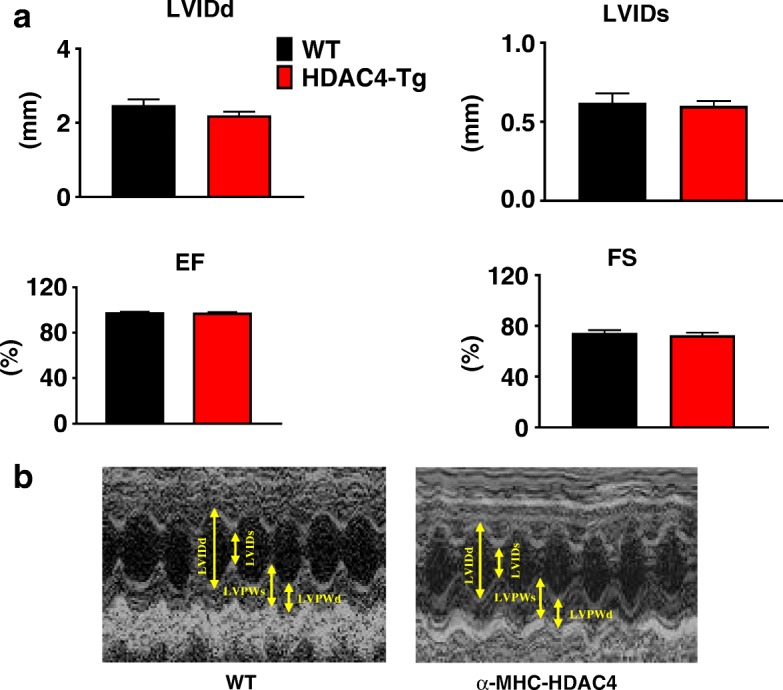
Myocardial function of α-MHC-HDAC4 mice and wild type littermates. **a** Echocardiographic measurements of cardiac function from α-MHC-HDAC4 mice and wild type mice. Values represent mean ± SE (*n* = 5/per group). EF: Ejection fraction; FS: Fractional shortening; LVID: left ventricle internal diameter; **b** Representative image of M-Mode of echocardiographic measurements

The HDAC4 proteins in the heart from MHC-HDAC4 levels were significantly higher than that of control wild type. In adult 2-month old mice, there was no differences in HDAC4 on other organs (Fig. 1a). The HDAC4 protein was increased in MHC-HDAC4 mice as compared to wild type (Fig. 1b). There is no difference in HDAC4 proteins in the other organs wild type mice (Fig. 1c). There is no obvious abnormality except for heart size at whole organ level (Fig. 1d). There were no significant difference cardiomyocytes and the heart weight-body weight ratio between MHC-HDAC and wild-type mice at two-month-old age, which is consistent with the myocyte sizes detected by WGA (Fig. 1e–g). HDAC4 activity was also increased in MHC-HDAC4-Tg mice as compared to wild type mice (Fig. 1h). Echocardiographic measurements show no differences in cardiac function as indicated by LVIDs, LVIDd, EF, and FS at two-month of age between wild type and MHC-HDAC4 mice (Fig. 2).

### Baseline ventricular functions prior to I/R

**Fig. 3:**
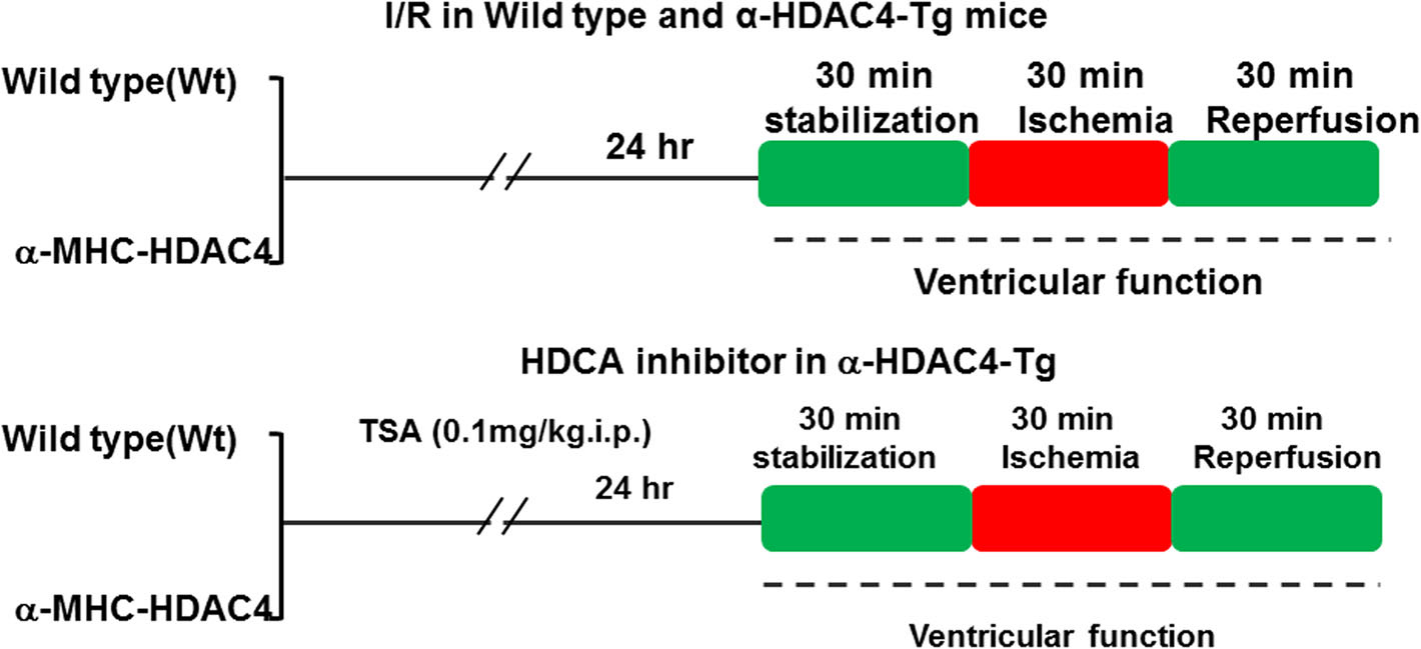
The experimental protocol for myocardial I/R. Wt: wild-type, TSA: trichostatin A; I/R: ischemia/reperfusion

Baseline functional parameters, including left ventricular developed pressure (LVDP), LV-dP/dt max LV-dP/dt min, heart rate, and coronary effluent were recorded among control wild type and HDAC4 transgenic mice before subjection to I/R. The experimental protocol for I/R was shown in Fig. 3. As shown in Table 1, there were no significant differences among the groups before ischemia.

**Table 1:** Baseline ventricular function in Langendorff hearts

Groups	LVDP mmHg	LV-dP/dt max mmHg/s	LV-dP/dt min mmHg/s	CF ml/min	Heart rate bmp
WT	80 ± 10	2563 ± 337	2773 ± 437	3.6 ± 0.3	392 ± 38
MHC-HDAC4	106 ± 15	2757 ± 417	2843 ± 2521	2.8 ± 0.4	368 ± 28
TSA + WT	115 ± 6	3126 ± 168	3066 ± 82	3.8 ± 0.5	349 ± 29
TSA+ α-MHC-HDAC4	94 ± 8	3394 ± 258	3237 ± 180	3.6 ± 0.3	380 ± 34

*LVDP* Left ventricular develped pressure, *CF* coronary effluent, *TSA* trichostatin A

No significant differences were found between the experimental groups for any of the functional parameters (*n* = 4–5/per group)

### Infarct size

**Fig. 4:**
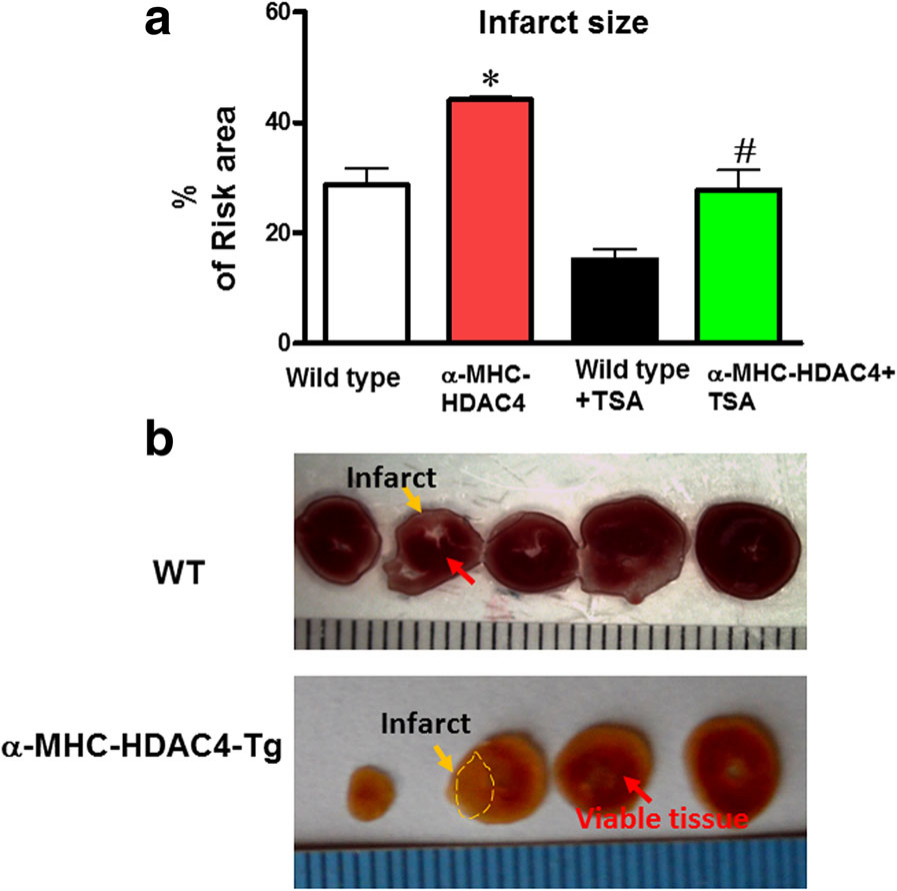
Myocardial infarct sizes in wild type and α-MHC-HDAC4 mice in response to I/R injury. **a** Myocardial infarction in wild type littermates and α-MHC-HDAC4 mice. **b** Representative image of infarct size. At the end of the experimental protocol as described in Methods, the hearts were sliced into 4–5 sections and stained with 2,3,5-triphenyltetrazolium chloride followed by fixation in formalin. Viable areas are stained brick red, whereas infarcted areas are gray or white. Values represent mean ± SE (*n* = 5/per group). *p < 0.05 vs wild-type mice ^#^*p* < 0.05 vs wild-type mice + TSA and α-MHC-HDAC4 mice

Myocardial infarct size, an index of irreversible myocardial injury, was measured. As shown in Fig. 4a, the infarct size following I/R in α-MHC-HDAC4 transgenic mice was (43.6 ± 0.6%) as compared with the wild type mice (28.6 ± 3.1%); the representative images are shown as Fig. 4b. This suggests that activation of HDAC4 increased infarct size in response to ischemia and reperfusion injury. However, following TSA treatment, the infarct size in α-MHC-HDAC4 transgenic mice group was reduced as compared to α-MHC-HDAC4 transgenic mice in absence of TSA treatment. The magnitude of infarct size in TSA-treated α-MHC-HDAC4 transgenic mice is still larger than that of TSA-treated wild type mice. The data suggest that HDAC4 overexpression in the heart increased myocardial infarct size. It also indicates that the increase in the infarct size in α-MHC-HDAC4 transgenic mice was blocked by inhibition of HDAC4 activity.

### Ventricular function recoveries following I/R

**Fig. 5:**
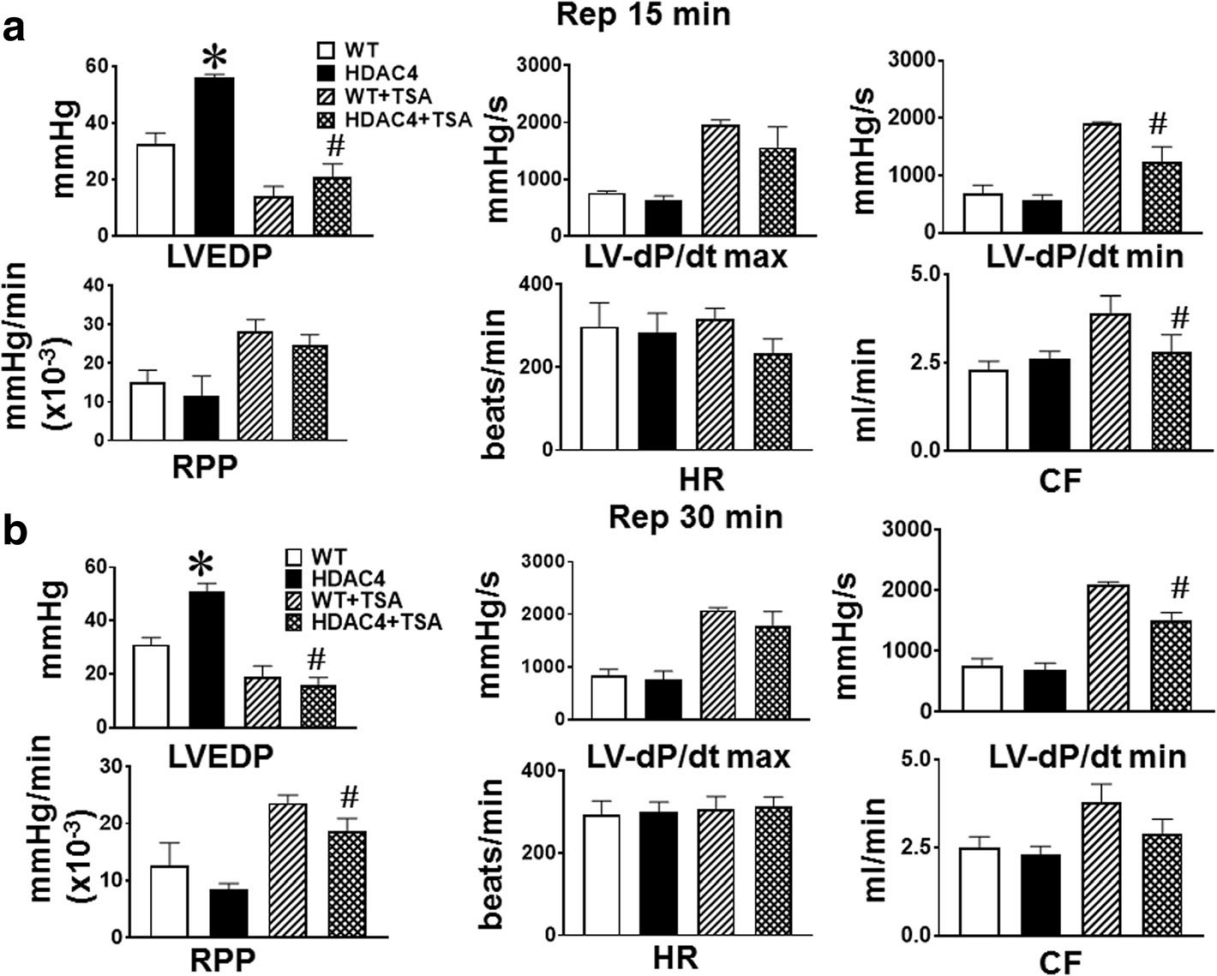
Post-ischemic ventricular functional recovery following ischemia and reperfusion at the different times. **a** At 15 min of reperfusion; **b** At 30 min of reperfusion. Left ventricular (LV) function was assessed in isovolumetric hearts. The measured parameters include LV systolic pressure developed pressure (LVDP), LV-dP/dt max, LV-dP/dt min, heart rate (HR) and coronary effluents (CF). Values represent mean ± SE (*n* = 4–5/per group), *p < 0.05 vs wild type mice; ^#^*p* < 0.05 vs. α-MHC-HDAC4 mice

As shown in Fig. 5, left ventricular functional recovery declined dramatically as compared with baseline. However, as compared with the wild-type control, the post-ischemic LVEDP demonstrated a remarked elevation in MHC-HDAC4 mice (*p* < 0.05 vs Wild type), which was also accompanied with the reduction of RPP recovery MHC-HDAC4 at both 15 min (Fig. 5a) and 30 min (Fig. 5b) of reperfusion. The coronary effluent and heart rate demonstrated a slight decline as compared to pre-ischemic stage, but showed no difference following I/R between wild type and MHC-HDAC4 mice (Table 1).

We investigated whether, TSA, an HDAC inhibitor, could target HDAC4 to attenuate the depression of ventricular functional recoveries following I/R in MHC-HDAC4 mice. We delivered trichostatin A at a dose of 0.1 mg/kg, which has been shown to be protective in I/R injury, to both wild-type and MHC-HDAC4 mice. TSA treatment blocked the depression of the recovery of left ventricular functional recovery in MHC-HDAC4 mice (Fig. 5a and b). Thus, HDAC4 overexpression exacerbates myocardial I/R injury, and this process is attenuated by therapeutic delivery of chemical HDAC4 inhibitor.

### Signaling mechanism of HDAC4 overexpression increased I/R injury

**Fig. 6:**
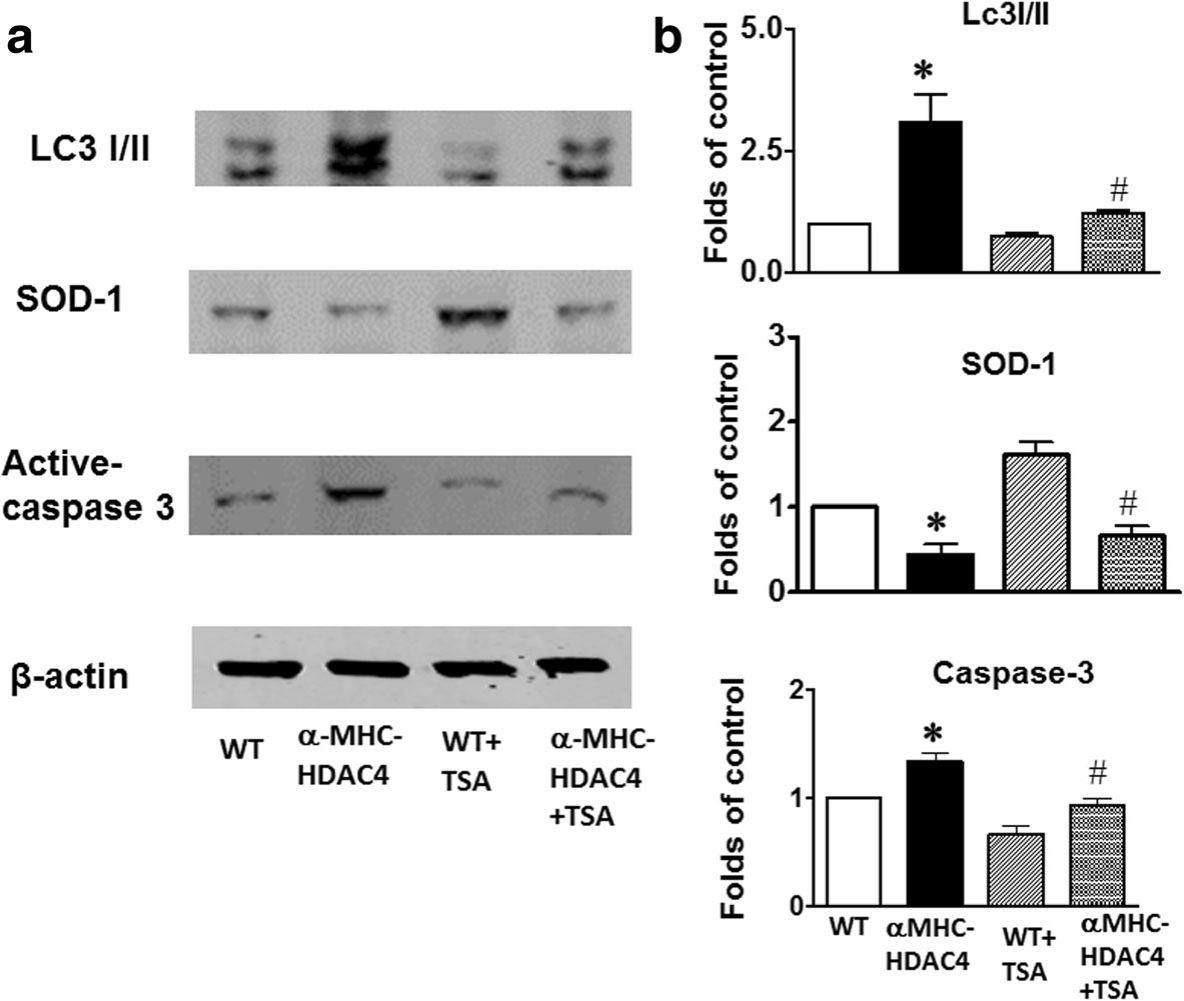
Active HDAC4 increased active caspase 3 and autophagy and decreased SOD1. **a** Western blot showing increased active caspase-3, decreased SOD-1, and increased LC-3I/II in the heart from HDAC4 transgenic mice; **b** Densitometric analysis of each protein level relative to wild type (*n* = 3 per group); Values represent mean ± SE, *p < 0.05 vs WT; ^#^*p* < 0.05 vs WT + TSA. SOD: Superoxide dismutase; LC3: autophagosome associated lipidated isoform LC3

**Fig. 7:**
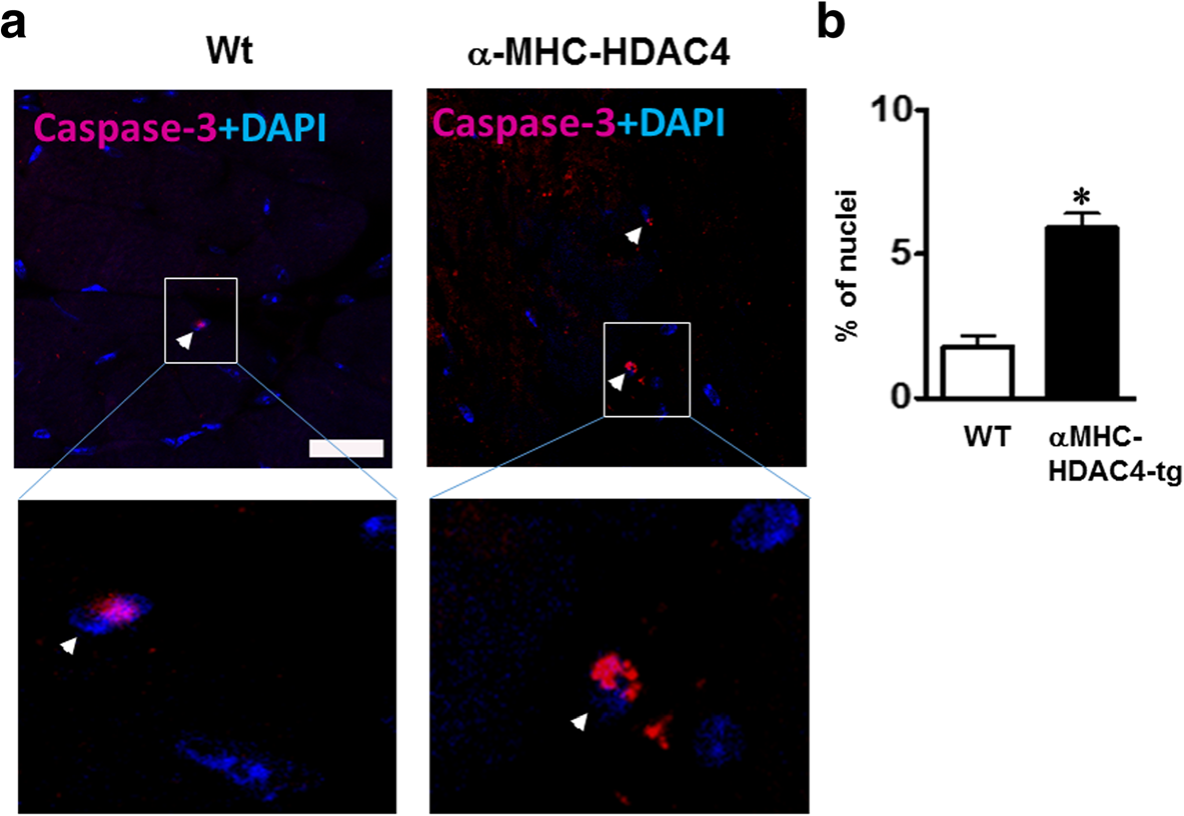
Immunostaining detecting active caspase-3 in myocardium. **a** Immunostaining showing the increase of active caspase-3 positive signals in HDAC4-Tg mice vs wild type littermates. **b** Percentage of active caspase-3 positive nuclei in the myocardium following I/R. Values represent mean ± SE (*n* = 3 per group); Scale bar = 10μm

It was noticed that the HDAC inhibition-induced reduction in cell death was correlated with the suppression in autophagy (Cao et al. 2011). Autophagy was evaluated by western blot detection of the autophagosome associated lipidated isoform LC3 (LC3-II). The LC3-II level, relative to autophagy abundance, was detected in the ischemic heart of wild type mice, but overexpression of HDAC4 resulted in a significant increase in LC3-II in the heart (Fig. 6a and b), but this increased LC3-II was attenuated by treatment of TSA. Furthermore, activation of HDAC4 also increased active-caspase 3 and decreased SOD-1 signals (Fig. 6a and b). TSA treatment attenuated the effect of HDAC4 on active caspase 3 and SOD-1 levels in the heart. The immunostaining was also confirmed by an increase in caspase-3 positive nuclei in the HDAC4-Tg heart (Fig. 7a and b).

### HDAC inhibition attenuated myocardial infarction in pig I/R model

As shown in Additional file 1: Figure S1a, there was no significant difference in the ratio of risk area/left ventricular area between control and TSA-treated groups. In contrast, as compared to the control group, TSA-treated group demonstrated a marked reduction in the ratio of infarct/risk area. Likewise, TSA treatment also manifested a significant reduction in infarct/left ventricular area. The representative histologic images are presented in Additional file 1: Figure S1b. The α-MHC-HDAC4 mice and wild type mice e results indicate that HDAC inhibition reduced myocardial infarction in the hearts exposed to I/R injury.

## Discussion

### Salient findings

In this study, we demonstrated that: 1) This is the first study to identify that overexpression of activated HDAC4, a major class II HDAC isoform in the heart exacerbates myocardial I/R injury, as indicated by the increase in infarct size and the reduction of myocardial function; 2) Over-expression of HDAC4-induced I/R injury was attenuated by delivery of HDAC inhibitor, TSA; 3) Furthermore, activated HDAC4 promoted I/R injury was associated with increased in autophagy, apoptosis and decreased SOD-1. These findings indicate that cardiac-specific activated HDAC4 reduces myocardial function and increases cardiac injury following I/R, which is associated with increased autophagy and apoptosis.

The roles of class II HDACs in cardiac development and hypertrophy were assessed in previous observations (Antos et al. 2003; Kee et al. 2006; Kong et al. 2006; Haberland et al. 2009; Granger et al. 2008). Class II HDAC4 only demonstrated a minimal enzymatic activity or lacked the activation in physiological condition. Importantly, cardiac injury or pathological stress resulted in enzymatic activation of HDAC4, suggesting that activated HDAC4 is more important for developing injury and serves as an effective target for potential therapy. In the present study, we created a cardiac-specific HDAC4 mouse model to provide the genetic and physiological evidence of HDAC4 in myocardial I/R. Our results indicated that specific activation of HDAC4 promotes myocardial injuries in the heart exposed to I/R, revealing that activated HDAC4 is crucial to modulate I/R injury. Previous reports indicated that the deletion of regular HDAC4 displayed premature ossification of developing premature bone and early onset chondrocyte hypertrophy (Zhang et al. 2012c). In addition, over-expression of HDAC2 had augmented hypertrophy, but HDAC2 deficiency prevented attenuated cardiac hypertrophy (Trivedi et al. 2007). Likewise, transgenic mice with mutant HDAC4 displayed greater left ventricular hypertrophy and a larger cross-sectional area of LV myocytes (Ago et al. 2008). Even though all of these studies points out the importance of HDACs in contribution to cardiac failure and development. There is no information to define the role of activated HDAC4 in in mediating cardiac ischemia and reperfusion injury.

HDAC inhibitors were tested in many disease models to achieve their therapeutic effects by antagonize enzymatic activity of various HDACs. Our previous works and other observations have demonstrated that HDAC inhibitors elicited cardioprotective effects against myocardial ischemic injury (Zhao et al. 2007; Zhang et al. 2012a; Zhang et al. 2012b; Zhang et al. 2010; Chen et al. 2011). Although HDAC inhibitors were largely included to investigate the function of HDAC4 in various pathological models, however, non-specific effects of HDAC inhibitor demonstrated the limitation of assessing the role of specific HDAC isoforms. In our previous study, HDAC inhibitor (TSA) caused the degradation of HDAC4 in addition to the inhibition of HDAC activity. It is likely that the magnitude of HDAC4 content in HDAC4-Tg mice could be reduced due to the degradation of HDAC4. Therefore, we sought to include trichostatin A to see whether the physiological function of cardiac HDAC4 would be affected in response to ischemia and reperfusion injury. We also used the same dose of TSA in wild type mice, which is consistent with our previous report showing that HDAC inhibitor demonstrated protective effects in wild type mice (Zhao et al. 2007). In this observation, delivery of HDAC inhibitor effectively blocked the deleterious effect of HDAC4 in I/R injury, revealing the importance of activated HDAC4 in contributing to I/R injury. This is supported by our previous studies in which cultured cardiomyocyte infected with HDAC4 increased hypoxic-induced cell damage, and trichostatin A antagonized the detrimental effect of HDAC4 in association with the reduction of HDAC4 (Du et al. 2015). Furthermore, HDAC4 was up-regulated in response to oxidant stress, and suppression of HDAC4 promoted embryonic stem cell-derived myogenesis and survival (Chen et al. 2011), implying that HDAC4 inhibition may function as a critical HDAC isoform attributable for the cardiac protective effect. More interestingly, we proceeded to define whether trichostatin A treatment could induce myocardial protection using a large animal model. Our results suggested that inhibition of HDAC protects the heart against ischemia/reperfusion injury in pig, as indicated by the reduction of myocardial infarct size. The finding provides a strong evidence demonstrating that HDAC inhibitor holds promise in developing a potential therapeutic strategy holding clinical implications in the future.

### Signaling pathway involving activated HDAC4-induced I/R injury

It was noticed that the HDAC inhibition-induced reduction in cell death was correlated with the suppression in autophagy (Cao et al. 2011). This change in autophagic activity was thought to be linked with a variety of pathological conditions and recognized to be involved in ischemia and reperfusion injury. Interestingly, a previous report indicated that HDAC inhibition attenuated cardiac hypertrophy in association with the suppression of autophagy, establishing a correlation between HDAC and induction of autophagy in response to cardiac hypertrophy (Cao et al. 2011). In agreement with this observation, our finding indicates that overexpression of HDAC4 resulted in an increase in autophagy, which was also attenuated by TSA treatment. In addition, myocardial ischemia and reperfusion injury demonstrates an over-expression of HDAC4 in the heart caused by the reduction in anti-oxidant enzymes (SOD) and increase in apoptosis (Wang et al. 2017), which were prevented by TSA treatment. Our results indicate that the specific activation of HDAC4 promotes myocardial injuries in the heart exposed to I/R, which is associated with reduction of SOD-1 and increased apoptosis. It is also interesting to see the effects of TSA on other class II HDACs in HDAC4 overexpression mice in response to I/R injury in the future.

## Conclusion

Our study provides direct evidence that active HDAC4 in the heart is crucial to promote myocardial I/R injury, and HDAC4-induced I/R injury can be attenuated by delivery of HDAC inhibitor. Furthermore, activated HDAC4-elicited cardiac injury was associated the increased autophagy, apoptosis and decreased SOD-1. Importantly, the studies provide new insight into understanding the molecular mechanism of active HDAC4 in I/R injury and hold promise in developing new therapeutic strategies to target active HDAC4.

## Additional file

Additional file 1: **Figure S1.** HDAC inhibition reduced myocardial infarct size in myocardial ischemia and reperfusion in pig. (TIF 3568 kb)

## Abbreviations

CF: Coronary effluents
EF: Ejection fraction
FS: Fractional shortening
HDAC4: Histone deacetylase 4
HR: Heart rate
I/R: Ischemia and reperfusion injury
LC3: Microtubule-associated protein light chain 3
LC3-II: Lapidated isoform of LC3, autophagy marker
LV: Left ventricular
LVDP: Left ventricular developed pressure
LVID: Left ventricle internal diameter
Rep: Reperfusion
SOD-1: Superoxide dismutase-1
TTC: Tetrazolium chloride
WGA: Wheat germ agglutinin
