# Supplementary figures and images for: Myocyte-specific overexpressing HDAC4 promotes myocardial ischemia/reperfusion injury
Source: Mol Med. 2018 Jul 17;24:37. doi: 10.1186/s10020-018-0037-2 (PMC6050730; doi:10.1186/s10020-018-0037-2)

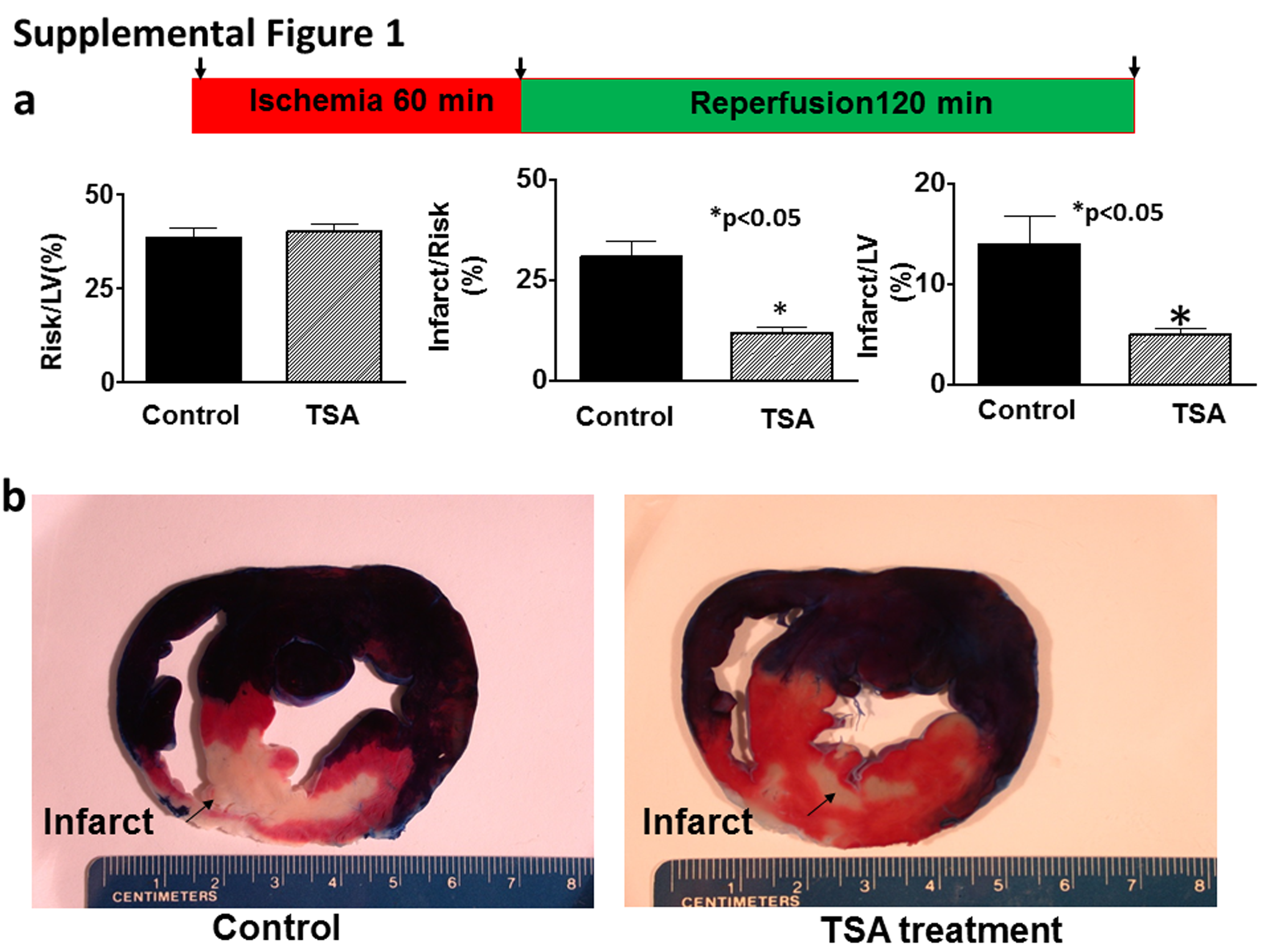

Supplement: Supplementary file 1 — Figure S1. HDAC inhibition reduced myocardial infarct size in myocardial ischemia and reperfusion in pig. (TIF 3568 kb) [file 10020_2018_37_MOESM1_ESM.tif]
